# An Unusual Case of Sternal Osteomyelitis and Purulent Mediastinitis After a Traumatic Sternal Body Fracture

**DOI:** 10.7759/cureus.74011

**Published:** 2024-11-19

**Authors:** Naib Chowdhury, Giuseppe Serena, Laura Velcu, Leonard Barrett, L. D. George Angus

**Affiliations:** 1 Department of Surgery, Nassau University Medical Center, East Meadow, USA

**Keywords:** blunt chest wall trauma, mediastinitis, sternal abscess, sternal fracture, sternal osteomyelitis

## Abstract

A 67-year-old female presented to the emergency department after falling on her chest. On initial presentation, her chest wall was tender to palpation with mild overlying ecchymosis. Initial imaging demonstrated a sternal body fracture with minimal retrosternal hematoma. On hospital day four, a fluctuant mass was appreciated over her sternum. A repeat CT scan demonstrated an abscess collection anterior and posterior to her sternum with underlying subcutaneous emphysema. The patient was taken to the operating room for incision and drainage with sternal body debridement. Pathology resulted in acute and chronic osteomyelitis and fluid culture with *methicillin-sensitive Staphylococcus aureus* (MSSA). Following infectious process resolution, the incision was closed with bilateral pectoralis advancement flaps. This case represents a rare complication of a relatively common traumatic injury, namely, a sternal fracture. In this scenario, due to the high mortality associated with mediastinitis, an immediate intervention and a multidisciplinary approach are cornerstones for optimal outcomes.

## Introduction

Sternal fractures occur in approximately 3% to 8% of older patients with blunt chest wall trauma [1,2]. Isolated sternal fractures are primarily managed conservatively and are typically without significant complications. However, in rare cases, retrosternal hematomas following sternal fractures can become a medium for bacterial seeding, leading to purulent mediastinitis and sternal osteomyelitis. Sternal osteomyelitis is an infection of the sternum, often originating from a retrosternal hematoma as in this patient population. Mediastinal infections are serious and potentially life-threatening, requiring prompt intervention to reduce mortality and morbidity. This case report describes the development of a mediastinal abscess and sternal osteomyelitis resulting from a retrosternal hematoma after blunt chest wall trauma.

## Case presentation

A 67-year-old female patient presented to the emergency department following a fall onto her chest at home. She was hemodynamically stable upon presentation and had a past medical history of attention-deficit/hyperactivity disorder, depression, hypertension, and alcohol dependence. The patient denied any history of IV drug abuse, dental procedures, or diabetes (with normal glycated hemoglobin (HbA1c) at presentation). On examination, she had mild ecchymosis and tenderness to palpation over the lower sternum. 

**Figure 1:**
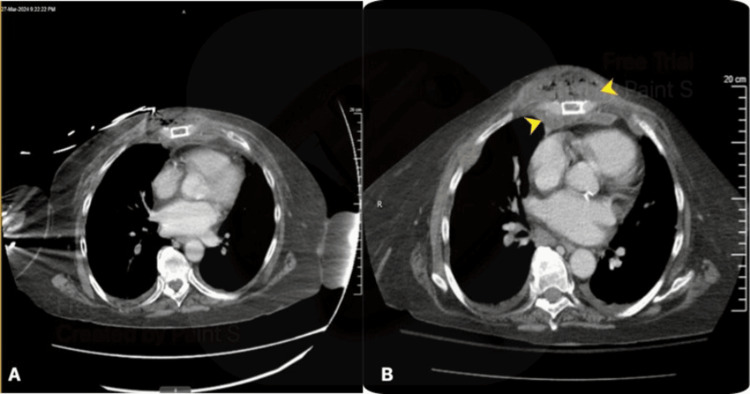
Computed tomography of the chest obtained on admission and preoperatively. Two axial images demonstrate the initial scan with a retrosternal hematoma (A) and subsequent preoperative abscess formation with extension into the mediastinum (B). Yellow arrows indicate the abscess formation with extension into the mediastinum.

Computed tomography (CT) with IV contrast revealed a fracture of the distal sternal body with associated soft tissue contusion, emphysema, and a small retrosternal hematoma (Figure 1a). Additional injuries included mildly displaced fractures of the right fifth and sixth anterior ribs. No evidence of hemothorax or pneumothorax was noted on imaging.

On hospital day four, the patient developed leukocytosis (22,000), and a fluctuant mass was noted at the mid-sternal region. Imaging revealed a 10 cm x 5 cm abscess anterior to the sternum, extending posteriorly into the anterior mediastinum (Figure 1b), with lucency over the sternal fracture suggestive of developing osteomyelitis. The patient had initially been started on IV antibiotics (ceftriaxone) upon admission for a symptomatic urinary tract infection (UTI), which were broadened (vancomycin and piperacillin/tazobactam) after the mediastinal abscess was identified.

**Figure 2:**
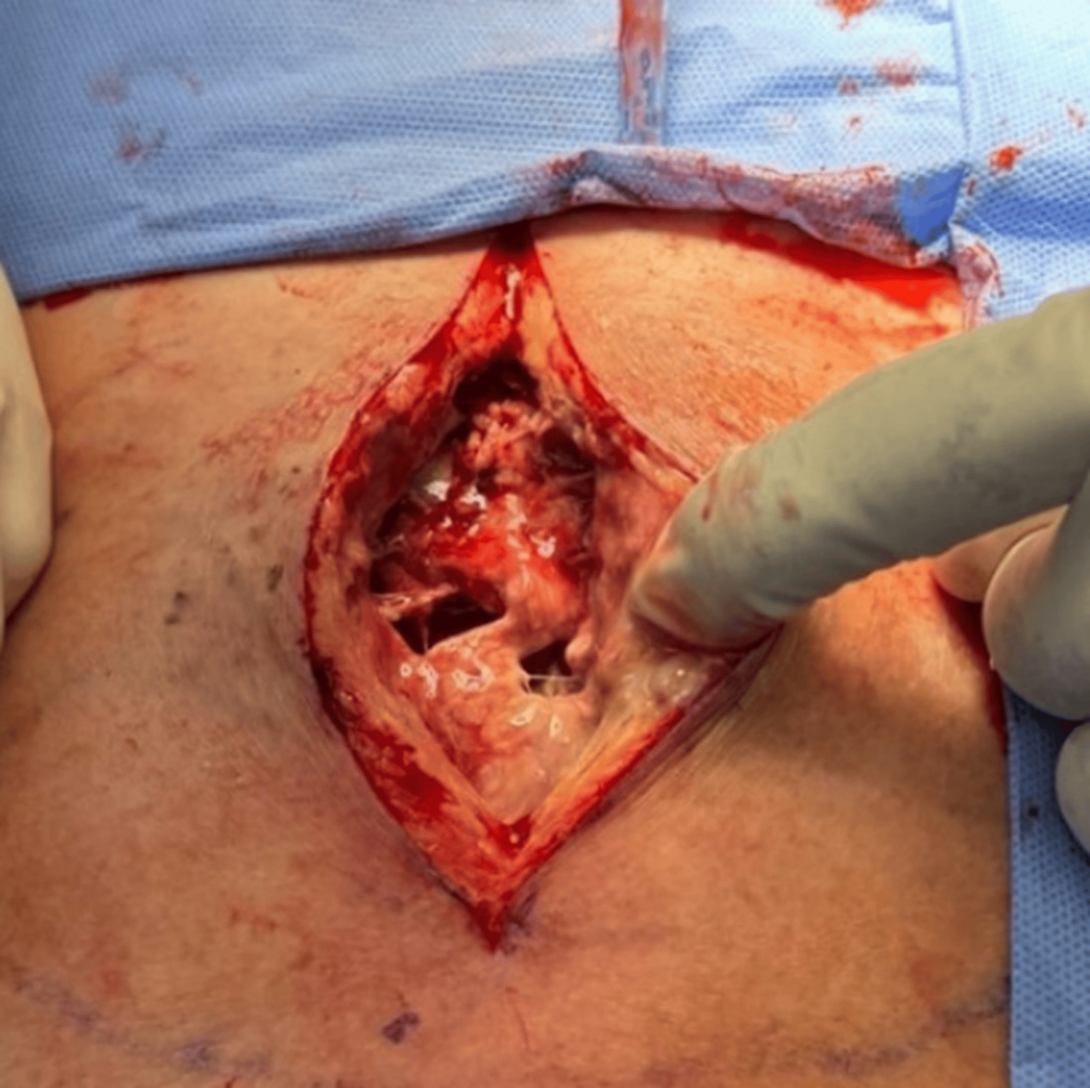
Intraoperative imaging of extensive purulence of the inferior sternum and extension of the infectious process over the mediastinum

**Figure 3:**
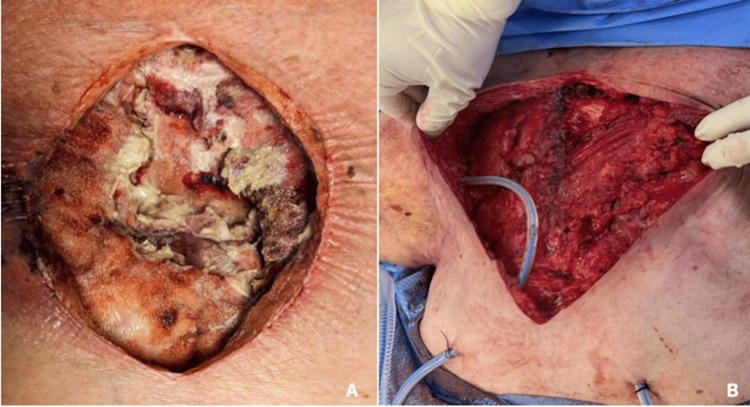
Wound after initial debridement (A) and one week after successful closure with bilateral pectoralis advancement flaps (B)

The patient underwent incision and drainage of the mediastinal abscess, sternal debridement, and partial sternotomy. Intraoperatively, extensive purulence was observed, with erosion of the lower sternum and extension of the infectious process into the mediastinum (Figure 2). The wound was initially left open and managed with daily packing changes, later transitioning to a wound vacuum-assisted closure (VAC) device (Figure 3a). It was successfully closed after one week using bilateral pectoralis advancement flaps (Figure 3b), with no further debridement or wash-out required.

Concurrently, the patient received intravenous antibiotics, tailored based on the fluid analysis from the intraoperative collection that identified *methicillin-sensitive Staphylococcus aureus *(MSSA), and sternal fragment analysis that confirmed acute and chronic osteomyelitis.

The patient remained hospitalized for drain management, daily hyperbaric treatment, and IV antibiotics. The sternal incision healed appropriately, and the patient was discharged to subacute rehabilitation on hospital day 29.

## Discussion

Osteomyelitis of the sternum and purulent mediastinitis secondary to blunt chest trauma is rare and may be unique because no source of bacteria could have inoculated the retrosternal hematoma [3]. This infection is typically associated with penetrating chest wall trauma, sternal instrumentation, or median sternotomy [4]. MSSA is the most common organism observed in sternal osteomyelitis with mediastinitis, which was also observed in our case. The most common factors associated with this complication are immunocompromised individuals, diabetes, IV drug users, and individuals who have had a catheter-related bloodstream infection, neither of which were observed in our patient, thereby making alcohol abuse the more likely contributing factor [5].

Bronchiectasis and urinary tract infection were present in our patient on admission; however, no current literature suggests that mediastinitis can arise from these disease processes. Malnutrition is another factor commonly associated with increased susceptibility and severity of infection. In a malnourished state, the integrity of the innate immune system, such as skin and mucosa, is compromised. Cell-mediated and humoral immune responses are impaired in malnourished individuals as well, leading to more frequent and more severe infective episodes. In older individuals, nutritional deficiency may turn relatively mild illnesses into life-threatening conditions [6]. In our case, the patient had a long history of alcohol abuse and presented with albumin and prealbumin at admission of 1.8 and 10.2, respectively, which could have predisposed the patient to bacterial seeding of the retrosternal hematoma. The arterial blood supply of the sternum is derived solely from the sternal branches of the internal mammary artery (IMA). There is no collateral blood supply to the cortical sternal bone, however, complete unilateral and limited bilateral IMA obliteration is unlikely to impair sternal blood supply to a significant degree. Only complete bilateral mobilization of the IMA at the subclavian arteries may render the entire sternum avascular [7]. Therefore, it is unlikely that impaired blood supply to the sternum predisposed our patient to sternal osteomyelitis.

The sternotomy wound was closed using bilateral pectoralis advancement flaps. The decision to use the pectoralis muscle flap for closure has remained consistent with recent literature due to the maintenance of reliable blood supply and decreasing operative time [8]. Furthermore, since the advent of the pectoralis muscle flap for closure, the average length of stay at the hospital has decreased from 19 days to 11 days [9,10]. Other options for the closure of similar wounds include utilizing the rectus abdominis muscle, omentum, or latissimus dorsi muscle [10]. However, the use of the omental flap requires access into the abdomen, increasing the risk of postoperative ventral hernia and increasing morbidity in a patient who is already in a stressed state [11]. The omental flap was not considered an optimum approach in our case. Other postoperative complications have remained fairly similar across all types of flaps, including hematoma, partial flap loss, or wound dehiscence [10].

Most reported cases of sternal osteomyelitis with mediastinitis, including our case, were treated with surgical exploration and debridement with long-term intravenous antibiotics for six weeks [12-14]. In our case, the inferior portion of the manubrium was completely eroded, and bilateral pectoralis advancement flaps were required to close the wound properly. Higher mortality rates, up to 42%, are seen with delayed diagnosis or treatment of acute mediastinitis [15]. The use of aggressive sternal debridement followed by delayed closure has decreased the mortality to less than 10% [10]. This highlights the importance of early diagnosis and aggressive treatment of purulent mediastinitis. To our knowledge, only nine previous cases of blunt chest wall trauma with sternal fracture complicated with sternal abscess and mediastinitis have been reported in the literature [5].

## Conclusions

To our knowledge, mediastinal abscess with sternal osteomyelitis is a rare complication of traumatic sternal fractures. A retrosternal hematoma can serve as a potential medium for bacterial seeding, leading to abscess formation and osteomyelitis. In this manuscript, we presented a patient successfully managed with incision and drainage, sternal debridement via sternotomy, long-term IV antibiotics, and bilateral pectoralis advancement flaps for wound closure. Although sternal fractures are relatively common in the trauma population, progression to sternal osteomyelitis and purulent mediastinitis is rare and associated with extremely high mortality. Immediate recognition and prompt surgical intervention are essential for optimal outcomes.
